# Oral Microbiota Characteristics in Relation to Different Dietary Patterns: A Systematic Review

**DOI:** 10.3390/nu18111717

**Published:** 2026-05-27

**Authors:** Alessandro Chiesa, Luigi Generali, Andrea Butera, Tommaso Filippini, Valentina Lanteri, Federica Veneri

**Affiliations:** 1 Department of Surgery, Medicine, Dentistry and Morphological Sciences with Transplant Surgery, Oncology and Regenerative Medicine Relevance (CHIMOMO), University of Modena and Reggio Emilia, 41124 Modena, Italy; luigi.generali@unimore.it (L.G.); valentina.lanteri@unimore.it (V.L.); 2 Department of Clinical, Surgical, Diagnostic and Pediatric Sciences, University of Pavia, 27100 Pavia, Italy; andrea.butera@unipv.it; 3 Department of Biomedical, Metabolic and Neural Sciences, University of Modena and Reggio Emilia, 41125 Modena, Italy

**Keywords:** oral microbiota, dietary patterns, mediterranean diet, vegan diet, microbiome diversity, taxonomic composition, oral health, dysbiosis

## Abstract

**Background:** Diet is a key modifiable factor influencing oral health and may shape the oral microbiota. While individual nutrients, especially free sugars, have been widely studied, the role of overall dietary patterns remains unclear. This systematic review aimed to evaluate the association between dietary patterns and oral microbiota in humans. **Methods:** PubMed/MEDLINE, Embase, and Web of Science were searched up to 18 March 2026. Studies assessing defined dietary patterns (Mediterranean, vegan, vegetarian, omnivorous) and oral microbiota using sequencing-based methods in healthy individuals were included. Due to heterogeneity in study design, dietary assessment, and microbiome analysis, a narrative synthesis was conducted. **Results:** Six studies (*n* = 448 participants) were included. Dietary patterns showed limited impact on overall microbiota structure, with no consistent changes in alpha and beta diversity. However, differences were observed at the taxonomic level. The Mediterranean diet was generally associated with a lower abundance of periodontopathogenic taxa. Plant-based and omnivorous diets showed distinct microbial profiles, particularly involving *Neisseria*, *Haemophilus*, *Prevotella*, and *Streptococcus*. Functional activity and metabolomic profiles appeared more sensitive to dietary variation than taxonomic composition alone. **Conclusions:** The oral microbiota appears relatively stable across dietary patterns, although diet may influence specific taxa and functional pathways relevant to oral health. The Mediterranean diet shows the most consistent association with beneficial microbial shifts. However, evidence is limited by heterogeneity and cross-sectional designs, highlighting the need for longitudinal and interventional studies.

## 1. Introduction

The oral microbiome represents a complex and dynamic ecological system, composed of diverse microbial communities organized in structured biofilms that interact with the host and contribute to maintaining oral homeostasis [1,2]. Under physiological conditions, a balanced microbiota supports colonization resistance and immune regulation, whereas its disruption, defined as dysbiosis, has been associated with the onset of oral diseases such as dental caries and periodontal diseases, as well as with several systemic conditions [3].

Among the environmental factors influencing the oral microbiome, diet plays a key role [2,4]. Dietary habits can modulate microbial communities by altering nutrient availability and local environmental conditions, including pH and substrate exposure [5,6]. In particular, there is strong evidence from human studies that fermentable carbohydrates, especially dietary sugars, serve as substrates for acidogenic bacteria, promoting acid production and enamel demineralization, thereby contributing to the development of dental caries [7]. Experimental human in vivo studies further support this ecological mechanism, showing that frequent sucrose consumption induces significant shifts in oral microbiota composition, with increased abundance of acidogenic species such as Streptococcus spp. [8]. Conversely, emerging evidence suggests that overall dietary patterns (not only single nutrients) may influence oral microbial ecology.

A healthy diet, in terms of variety, amount and frequency of food consumption, has a profound impact on physical and oral health, well-being and overall quality of life [9]. Alongside, healthier dietary patterns, such as Mediterranean or plant-based diets, have been associated with a reduction in pathogenic species and more favorable microbial profiles, potentially mediated by anti-inflammatory and metabolic effects [5]. These findings support a broader ecological perspective, in which diet contributes to shaping microbial balance rather than acting through isolated components.

In line with this paradigm shift, nutritional epidemiology has increasingly moved from single-nutrient approaches to the study of dietary patterns, which better reflect real-life eating behaviors and account for synergistic interactions among dietary components [10]. While this approach has been extensively applied to gut microbiota research, its application to the oral microbiome remains limited and characterized by inconsistent findings [11,12]. Additionally, the recent literature has suggested bidirectional interactions between the oral and gut microbiota, where perturbations in one microbial community influence the other, leading to increased risk of disease development [13,14]. Compared with the gut microbiome, the oral microbiome appears to exhibit greater temporal stability and resilience to dietary perturbations, likely due to the unique ecological characteristics of the oral cavity, including salivary flow, host antimicrobial factors, and continuous environmental exposure [15,16,17].

Current evidence indicates that the oral microbiome is characterized by a relatively stable core community, commonly including genera such as *Streptococcus*, *Neisseria*, *Prevotella*, and *Veillonella*, which are consistently detected across individuals [2]. However, dietary patterns may still induce ecological shifts at a more subtle level [18,19]. Indeed, available data suggest that diet-related effects are more likely to affect the relative abundance of specific taxa and microbial functions rather than global diversity metrics [1,20].

From a clinical perspective, this is highly relevant. Diet-induced microbial shifts may contribute to disease risk not necessarily through major compositional changes, but through functional alterations in metabolic pathways, including carbohydrate metabolism and acid production. For example, metagenomic evidence indicates that dietary sugar intake is associated with increased abundance of cariogenic microorganisms and enhanced microbial pathways related to sugar metabolism [21]. These observations support growing interest in understanding whether long-term dietary patterns may contribute to subtle ecological or functional shifts within the oral microbiome that may play a central role in disease development. Alterations in oral microbial composition and function have been associated not only with oral diseases, but also with systemic conditions such as cardiovascular disease, diabetes, obesity, and inflammatory disorders [22].

Despite growing interest in this topic, the current body of evidence remains limited and controversial. Studies differ substantially in dietary assessment methods, microbiome sampling sites (e.g., saliva, plaque, calculus), and sequencing techniques, limiting comparability. Moreover, most available studies are observational and cross-sectional, precluding causal inference and increasing susceptibility to confounding factors such as oral hygiene, smoking, and body mass index.

Taken together, these limitations highlight the need for a comprehensive synthesis of the available evidence. Therefore, the aim of the present systematic review was to critically evaluate the association between dietary patterns and oral microbiota characteristics in humans, with particular focus on microbial diversity, taxonomic composition, and functional implications.

## 2. Materials and Methods

### 2.1. Protocol and Registration

This systematic review was conducted in accordance with the PRISMA 2020 (Preferred Reporting Items for Systematic Reviews and Meta-Analyses) guidelines and followed methodological recommendations from SWiM (Synthesis Without Meta-analysis) and STORMS (Strengthening The Organization and Reporting of Microbiome Studies) frameworks [23,24,25]. The review protocol was prospectively registered in PROSPERO (CRD420251207041).

The review aimed to synthesize available evidence on the association between dietary patterns and oral microbiota characteristics in humans. Given the methodological heterogeneity across included studies, particularly in dietary exposure assessment, oral sampling sites, and microbiome sequencing approaches, a quantitative meta-analysis was deemed inappropriate, and a structured narrative synthesis was performed.

### 2.2. Search Strategy and Study Selection

A comprehensive literature search was conducted in the following electronic databases: PubMed/MEDLINE, Embase and Web of Science (WOS).

The search strategy combined controlled vocabulary (e.g., MeSH, Emtree) and free-text terms related to oral microbiota and dietary patterns (including Mediterranean, vegan, vegetarian, and other diet types). Details on the search strings used are reported in Table 1.

The search covered all records from database inception to 18 March 2026, with no date restrictions. Only studies published in English were considered.

All records were imported into the Rayyan web tool, and duplicates were identified and removed through the related algorithm [26]. The screening of titles, abstracts, and full texts for inclusion was performed independently by two authors (FV and AC). Disagreements were resolved through discussion and, when necessary, consultation with a third reviewer (LG).

Eligibility criteria were defined using the PECO(S) framework. Studies conducted in healthy human participants of any age (P) were included. The exposure of interest (E) was long-term dietary patterns, including but not limited to Mediterranean diet, vegan and vegetarian diets, omnivorous dietary patterns, and other clearly defined dietary clusters. Studies focusing exclusively on single nutrients or dietary components were excluded to maintain a focus on specific dietary patterns. Eligible comparators (C) included: different dietary patterns, different levels of adherence (e.g., Mediterranean diet scores); baseline vs. post-intervention conditions in longitudinal studies. The considered outcomes (O) included oral microbiota characteristics, specifically Alpha diversity (e.g., Shannon, Simpson indices), Beta diversity (e.g., Bray–Curtis, UniFrac distances) and taxonomic composition (focusing on genus-level relative abundance). Additional secondary outcomes, such as oral clinical outcomes (e.g., caries indices, periodontal parameters), were considered when reported. Only studies employing validated microbiome analysis methods, such as sequencing-based microbiome analyses (e.g., 16S rRNA gene sequencing or metagenomics), were included. With regard to the eligible study designs (S), randomized controlled trials (RCTs), non-randomized interventional studies and observational cohort, case–control, and cross–sectional studies were considered for inclusion. Case reports, case series, conference abstracts, and theses were excluded. Additionally, studies were excluded if they were conducted in animals or in vitro, oral microbiota was not assessed, diet was only included as a covariate without group comparison, participants had systemic conditions strongly affecting oral microbiota (e.g., diabetes, cancer), unless clearly controlled, or recent antibiotic use was reported. Studies were also excluded if relevant specific information was not explicitly reported.

### 2.3. Data Extraction

Data extraction was performed independently by two reviewers (AC and FV) using a standardized data extraction form. When available, extracted variables included: study characteristics (first author, year of publication, country; study design), population characteristics (sample size, age, sex distribution; body mass index (BMI) and lifestyle factors—smoking, alcohol use, oral hygiene practices), dietary exposure (type of dietary pattern—e.g., mediterranean, vegan, omnivorous; assessment method—e.g., food frequency questionnaires, adherence scores, intervention protocols), microbiome assessment (sampling site—e.g., saliva, dental plaque, dental calculus; sampling conditions—e.g., fasting state, timing relative to oral hygiene; sequencing methodology—e.g., 16S rRNA gene sequencing, targeted regions where reported), outcomes (alpha diversity indices—e.g., Shannon, Simpson, Chao; beta diversity metrics—e.g., Bray–Curtis, UniFrac distances; taxonomic composition with emphasis on genus-level relative abundance; oral clinical outcomes—e.g., periodontal indices, caries measures), and confounding factors (variables considered or adjusted for in the analysis—e.g., smoking, BMI, oral hygiene). Discrepancies in data extraction were resolved through consensus.

### 2.4. Data Analysis

Due to substantial heterogeneity across studies in terms of dietary exposure definitions, microbiome sampling and outcome reporting, a quantitative meta-analysis was not feasible. A structured narrative synthesis was conducted following SWiM guidance [23].

Studies were grouped according to dietary pattern (e.g., Mediterranean diet, plant-based diets, omnivorous diets).

The synthesis focused on the following predefined outcomes: Alpha diversity (focusing on Shannon index); Beta diversity (such as Bray–Curtis and UniFrac distances); Operational Taxonomic Units (OTUs) and relative abundance of key genera, such as *Streptococcus*, *Prevotella*, *Neisseria*, *Porphyromonas*, *Veillonella*.

Where appropriate, results were summarized in terms of direction of effect (increase, decrease, no changes). Oral clinical outcomes were analyzed descriptively when reported.

Interpretation of findings considered methodological differences, dietary assessment methods and potential residual confounding.

### 2.5. Quality and Risk of Bias Assessment (RoB)

The methodological quality of included studies was assessed using the NIH Quality Assessment Tool (NIH—National Institute of Health), adapted to the specific study designs (cross-sectional and interventional) [27].

The following domains were evaluated: clarity of the research question, definition and representativeness of the study population, assessment of dietary exposure, microbiome assessment methodology, control of confounding variables, and sample size adequacy. The detailed criteria adopted for the risk of bias assessment are reported in Supplementary Table S1.

Items related to temporality and follow-up were not applicable to most included studies and were therefore excluded from the assessment.

Each domain was rated as low, moderate, or high risk of bias, and an overall risk of bias judgment was assigned. The results of the RoB assessment were elaborated with the dedicated Robvis tool [28].

## 3. Results

### 3.1. Study Selection

**Figure 1:**
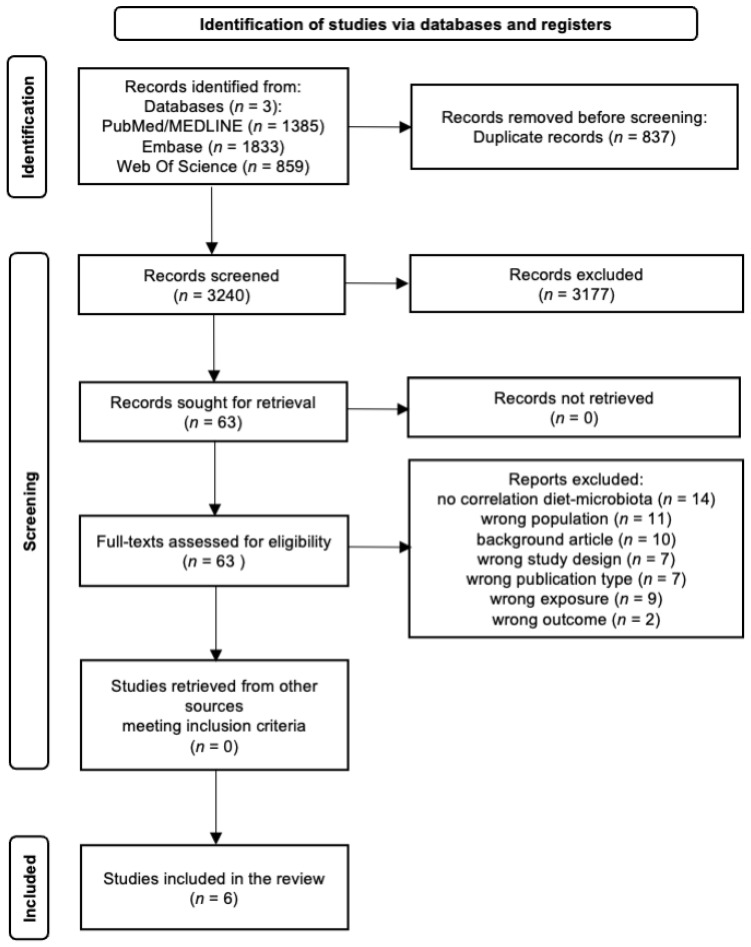
PRISMA 2020 flowchart [25].

The literature search identified 4077 records, of which 3240 remained after duplicate removal. Following title and abstract screening, 63 articles were assessed for full-text eligibility. Ultimately, 6 studies met the inclusion criteria and were included in the qualitative synthesis. PRISMA flowchart is displayed in Figure 1.

### 3.2. Characteristics of Included Studies

The included studies were published between 2014 and 2024 and were conducted in Italy (n = 4), Denmark (n = 1), and China (n = 1). Five studies had a cross-sectional design, while one was an interventional study evaluating the effects of a Mediterranean diet intervention over 8 weeks [29]. The total sample size across studies was 448 participants, with individual study populations ranging from 36 to 161 participants. Most participants were healthy adults, although one study included overweight/obese individuals undergoing dietary intervention.

The dietary exposures investigated included mediterranean diet (MD) (n = 3 studies), vegan/vegetarian diets (n = 4 studies), omnivorous diets (n = 4 studies), and specific omnivore subtypes (seafood vs. red meat) (n = 1 study). Dietary assessment methods varied, including self-reported questionnaires and structured dietary interventions.

As for microbiome assessment, saliva was the most commonly analyzed sample (5 out of 6 studies). One study evaluated the dental calculus microbiome, representing a distinct oral niche [30].

All studies employed 16S rRNA gene sequencing, although methodological details, such as targeted regions, were inconsistently reported.

Sampling protocols were heterogeneous; some studies standardized saliva collection (e.g., fasting conditions), while others did not report timing relative to meals or oral hygiene.

The main characteristics of the included studies are displayed in Table 2.

To facilitate the interpretation of the heterogeneous findings across studies, a direction-of-effect summary is reported in Table 3 to qualitatively synthesize the reported associations between dietary patterns and oral microbiota outcomes, including alpha diversity, beta diversity, taxonomic composition, and functional/metabolomic profiles.

### 3.3. Results of the Quality and Risk of Bias Assessment

Most studies (5 out of 6) were rated as having a moderate risk of bias [11,12,29,30,31]. Across studies, the following domains were most frequently associated with potential bias: dietary exposure assessment (often based on self-reported questionnaires without validated adherence scoring); confounder control (incomplete adjustment for key variables such as oral hygiene, smoking, BMI, and antibiotic use); sample size (several studies had moderate sample sizes, limiting statistical power).

**Figure 2:**
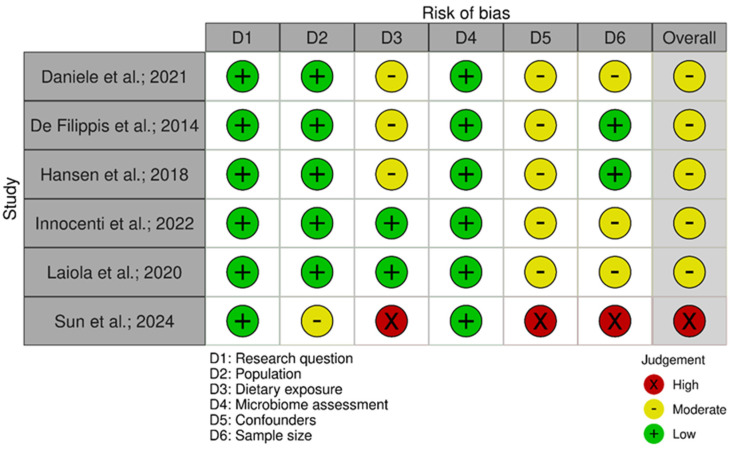
Quality assessment of the included studies according to the NIH quality assessment tool (Daniele et al.; 2021 [31]; De Filippis et al.; 2014 [11]; Hansen et al.; 2018 [12]; Innocenti et al.; 2022 [30]; Laiola et al.; 2020 [29]; Sun et al.; 2024 [32]. Laiola et al. 2020 [29]: Interventional study: the domains and their evaluation were adapted for this study, according to the specific study design.

One study was judged at high risk of bias due to limited confounder control and small sample size [32]. The details of the quality assessment are reported in Figure 2.

Notably, the predominance of cross-sectional designs limits causal inference and increases susceptibility to residual confounding. These limitations were explicitly considered during data synthesis and interpretation.

### 3.4. Synthesis of Findings

Findings regarding alpha diversity were heterogeneous but largely consistent in showing minimal dietary impact. No significant differences were observed between dietary patterns (vegan, vegetarian, omnivorous, Mediterranean) in several studies. One study reported differences in the Simpson diversity index, although richness (Chao index) remained unchanged [32]. Overall, alpha diversity appears relatively stable across dietary patterns, suggesting resilience of oral microbial richness and evenness.

Beta diversity analyses similarly indicated limited influence of diet on global microbial community structure. No significant clustering by dietary pattern was observed in multiple studies using UniFrac-based metrics. Mediterranean diet intervention did not significantly alter beta diversity. However, one study identified subtle differences in beta diversity between vegans and omnivores, primarily driven by differences in taxa abundance rather than community membership [12].

These findings highlight that inter-individual variability likely outweighs dietary effects on overall microbiota structure.

As for the taxonomic composition, across all studies, a stable core oral microbiota was consistently identified, dominated by genera such as *Prevotella*, *Streptococcus*, *Neisseria*, *Veillonella*, and *Haemophilus*. These taxa were present regardless of the dietary pattern, supporting the concept of oral microbiome ecological stability.

Despite overall stability, specific taxa exhibited diet-related variations. With regard to the Mediterranean diet, an increased abundance of *Prevotella* and *Neisseria subflava* was observed, along with a reduction in periodontopathogenic species (*Porphyromonas gingivalis*, *Treponema denticola*, *Prevotella intermedia*) following dietary intervention [29]. When comparing vegan vs. omnivorous diets, vegan participants displayed a higher abundance of *Neisseria*, *Haemophilus*, and *Rothia*. Conversely, an omnivore diet was associated with a higher abundance of *Prevotella* and *Streptococcus*.

Overall, vegetarian diets appeared to be associated with *Neisseria*–dominant profiles, while Meat-based diets were associated with *Streptococcus*–dominant profiles.

However, some studies reported no significant taxonomic differences, reinforcing the inconsistency across findings [32].

Regarding functional and metabolomic findings, data were limited but suggested that metabolomic profiles are more sensitive to dietary patterns than taxonomic composition. Additionally, differences in metabolic pathways (e.g., lipid, amino acid, carbohydrate metabolism) were observed across dietary groups. This indicates that functional adaptations may occur without major compositional shifts.

Noteworthily, one pilot study investigated dental calculus as the sampling site for microbiome, and identified a potential influence of diet on microbial composition of this niche, suggesting the utility of dental calculus as a long-term dietary biomarker. However, evidence remains limited due to the exploratory nature of this study.

Overall, across the included studies, the oral microbiota demonstrated high compositional stability across dietary patterns, particularly in terms of diversity metrics. Nevertheless, specific taxa and functional pathways appear responsive to dietary exposures, with the Mediterranean diet showing the most consistent association with a reduction in periodontopathogenic bacteria. The evidence base is limited by heterogeneity and predominance of observational designs, precluding definitive conclusions regarding causality.

## 4. Discussion

The present systematic review provides a comprehensive synthesis of the available evidence on the relationship between dietary patterns and oral microbiota characteristics. Overall, the findings suggest that the oral microbiome exhibits a high degree of compositional stability across different dietary patterns, particularly as shown by diversity indexes. However, more subtle but potentially clinically relevant differences emerge at the level of specific taxa and microbial functions, indicating that diet may act as a modulatory factor rather than a primary determinant of oral microbial ecology [33,34].

One of the most consistent observations across the included studies is the limited impact of diet on alpha and beta diversity. Most studies reported no significant differences in richness or overall community structure between dietary groups, regardless of whether comparisons involved vegan, vegetarian, omnivorous, or Mediterranean dietary patterns [11,12,31]. This aligns with the concept of a resilient core oral microbiome, dominated by genera such as *Streptococcus*, *Prevotella*, *Neisseria*, and *Veillonella*, which appear to be conserved across individuals and dietary exposures [35].

From an ecological standpoint, this stability may reflect the strong host-driven selection pressures in the oral cavity, including salivary flow, immune factors, and epithelial shedding, which may constrain large-scale microbial shifts [35]. In contrast to the gut microbiome, where diet is a dominant driver of microbial composition, the oral microbiome appears to be less sensitive to long-term dietary variation at a community-wide level [36,37].

Nevertheless, several studies identified diet-associated differences in specific taxa, suggesting that dietary patterns may influence relative abundance rather than the presence/absence of microbial species. In particular, the Mediterranean diet was consistently associated with a reduction in periodontopathogenic bacteria, including *Porphyromonas gingivalis*, *Treponema denticola*, and *Prevotella intermedia* [29,30,31]. This finding is biologically plausible and clinically relevant, as these taxa are key members of dysbiotic periodontal communities and have been implicated in systemic inflammatory pathways [38].

Similarly, plant-based diets were associated with higher abundance of genera such as *Neisseria* and *Haemophilus*, whereas omnivorous diets showed higher abundance of *Prevotella* and *Streptococcus* [12,32]. However, these findings were not consistent across all studies, highlighting the context-dependent nature of diet–microbiome interactions.

An important emerging insight is that functional and metabolomic changes may be more sensitive to dietary patterns than taxonomic composition. Studies incorporating metabolomic analyses demonstrated that dietary groups could be clearly distinguished based on metabolic profiles, even in the absence of major compositional differences [11,31]. This supports the concept that functional redundancy within the oral microbiome allows metabolic adaptation without substantial taxonomic restructuring, a phenomenon increasingly recognized in microbial ecology [39].

These findings are consistent with previous research suggesting that the oral microbiome is characterized by high inter-individual variability and temporal stability, with relatively modest responsiveness to environmental factors compared to other body sites [40]. The relatively limited diet-associated taxonomic variation observed in the oral microbiome contrasts with findings from gut microbiome research, where dietary patterns are recognized as major determinants of microbial composition and diversity [41,42]. Several ecological factors may contribute to this difference. Unlike the gut environment, which is continuously exposed to dietary substrates under relatively stable anaerobic conditions, the oral cavity is characterized by frequent environmental perturbations, including salivary flow, fluctuations in pH and oxygen availability, mechanical clearance, host antimicrobial peptides, and oral hygiene practices [1,17]. These factors may contribute to greater ecological resilience and temporal stability of the oral microbiome, limiting large-scale taxonomic restructuring in response to diet alone.

At the same time, the observed diet-related differences in specific taxa and functional pathways align with the broader ecological model of oral disease, in which shifts in microbial activity rather than composition drive dysbiosis [43,44]. In this context, the concept of functional redundancy may be particularly relevant. Different microbial taxa may perform overlapping metabolic functions, allowing adaptation to dietary exposures through changes in microbial activity and metabolic pathways rather than through major shifts in community composition [45]. This interpretation is consistent with the observation that several included studies reported more evident differences in metabolomic or functional profiles than in global diversity metrics.

Interestingly, several taxa frequently identified as part of the resilient core oral microbiome, including *Streptococcus*, *Prevotella*, *Veillonella*, and *Neisseria*, are also commonly detected in the gastrointestinal microbiome [41,46]. Although their ecological roles differ according to the anatomical niche, this overlap may reflect broader host-associated microbial networks and shared metabolic capabilities across mucosal microbial ecosystems [46].

Importantly, the observed reduction in periodontopathogens in association with Mediterranean diet adherence supports the hypothesis that anti-inflammatory dietary patterns may promote a more eubiotic oral environment, potentially contributing to disease prevention. This is in line with growing evidence linking diet quality to periodontal health and systemic inflammation [1,47].

The interpretation of these findings must be considered in light of several important methodological limitations, both at the study level and at the level of the evidence base.

First, the predominance of cross-sectional study designs represents a major limitation. Cross-sectional analyses do not allow for temporal or causal inference and are particularly vulnerable to reverse causation [48]. It is therefore unclear whether observed microbial differences are a consequence of dietary patterns or reflect underlying host or lifestyle characteristics.

Second, dietary exposure assessment was generally suboptimal. Most studies relied on self-reported dietary questionnaires without validated adherence scores or quantitative intake data. In some cases, incomplete dietary information further limited interpretability. Given the complexity of dietary patterns, this introduces a substantial risk of misclassification bias, potentially attenuating true associations [49].

Third, confounding remains insufficiently addressed across studies. Key determinants of oral microbiota composition, such as oral hygiene practices, smoking, alcohol consumption, recent antibiotic use, and periodontal status, were inconsistently reported and rarely controlled for in statistical analyses. This is particularly relevant in oral microbiome research, where local environmental factors may exert stronger effects than systemic exposures [50].

Fourth, heterogeneity in microbiome methodologies further limits comparability. Although all studies employed 16S rRNA gene sequencing, there was variability in the targeted hypervariable regions, sequencing platforms, bioinformatic pipelines, and taxonomic databases.

These methodological differences can significantly influence taxonomic resolution and relative abundance estimates, complicating cross-study comparisons [51].

Fifth, sampling protocols were not standardized. Variations in saliva collection (e.g., fasting vs. non-fasting, timing relative to oral hygiene) can introduce substantial variability in microbial profiles [52]. Only a subset of studies controlled for these factors, limiting internal validity.

Sixth, sample sizes were generally moderate, and in some cases small, reducing statistical power and increasing the risk of type II error. This may partly explain the inconsistent findings across studies.

Finally, the inclusion of different oral niches (saliva vs. dental calculus) adds an additional layer of heterogeneity [15]. While saliva reflects a composite microbial signal, dental calculus represents a more stable, long-term biofilm. The limited evidence on non-salivary niches precludes firm conclusions regarding their relationship with diet.

Despite these limitations, this review has several strengths. It represents one of the first attempts to systematically synthesize evidence on dietary patterns (rather than single nutrients) and oral microbiota, aligning with contemporary approaches in nutritional epidemiology [53]. The use of structured frameworks and a critical risk of bias assessment enhances transparency and methodological rigor. Furthermore, the focus on both taxonomic and functional outcomes provides a more comprehensive understanding of diet–microbiome interactions [43,44].

The findings of this review suggest that dietary patterns may influence oral health not through large-scale microbial shifts, but through modulation of specific taxa and metabolic functions, particularly those involved in inflammation and carbohydrate metabolism. This has potential implications for preventive strategies, highlighting diet as a modifiable factor in oral ecosystem balance [1,33]. However, the current evidence is insufficient to support definitive clinical recommendations. Future research should prioritize longitudinal and interventional study designs, standardized and validated dietary assessment tools, rigorous control of confounding variables (such as oral hygiene, smoking, BMI, and medication use), integration of multi-omics approaches (metagenomics, metabolomics, transcriptomics), and investigation of site-specific microbiomes (e.g., plaque vs. saliva) or standardized oral sampling sites. Such approaches will be essential to identify causal relationships and better understand the mechanisms linking diet, oral microbiota, and disease. Beyond oral diseases, increasing evidence suggests that alterations in oral microbial ecology may also contribute to systemic inflammatory processes and chronic diseases [54]. Although the studies included in this review were not designed to establish causal relationships, some of the identified diet-associated microbial shifts involved taxa associated with inflammatory and periodontal pathways. This observation further supports the hypothesis that dietary patterns could influence host health not only through direct nutritional mechanisms but also through modulation of host-associated microbial ecosystems [2].

## 5. Conclusions

In conclusion, the currently available evidence suggests that the oral microbiota is relatively stable across different dietary patterns, with limited and inconsistent effects on global microbial diversity. Some studies reported differences in the relative abundance of specific taxa and microbial functional profiles, particularly in association with Mediterranean and plant-based diets. However, the interpretation of the available evidence is limited by the predominance of observational and cross-sectional study designs, which restrict causal inference and remain susceptible to residual confounding. In addition, considerable heterogeneity in dietary assessment methods, oral sampling sites, sequencing protocols, and statistical adjustment strategies limits comparability across studies. Therefore, no definitive conclusions can currently be drawn regarding the impact of dietary patterns on oral microbiota composition. Further well-designed longitudinal and interventional studies using standardized methodologies are needed to clarify these associations.

## Figures and Tables

**Table 1:** Details of literature search strategies in online databases.

Database	Search String
PubMed/ MEDLINE	(oral microbiota) OR (oral microbiome) OR ((microbiota) AND (oral)) OR ((microbiome) AND (oral)) OR (“Mouth”[Mesh] AND “Microbiota”[Mesh]) AND ((diet) OR “diet”[Mesh] OR “Feeding Behavior”[Mesh] OR “Food Preferences”[Mesh] OR (vegan) OR (vegetarian) OR (plant-based) OR (Mediterranean) OR (DASH) OR (ultra-processed) OR (sugar) OR (low-carbohydrate) OR (ketogenic) OR (high-protein) OR (low-fat)) AND humans[MH]
Embase	(‘oral microbiota’/exp OR ‘oral microbiome’/exp OR (‘oral’ AND ‘microbiota’) OR (‘oral’ AND ‘microbiome’)) AND (‘diet’/exp OR ‘feeding behavior’/exp OR ‘food preferences’/exp OR ‘vegan’/exp OR ‘vegetarian’/exp OR ‘plant-based’/exp OR ‘Mediterranean’/exp OR ‘DASH’/exp OR ‘ultra-processed’/exp OR ‘sugar’/exp OR ‘low-carbohydrate’/exp OR ‘ketogenic’/exp OR ‘high-protein’/exp OR ‘low-fat’/exp) AND ‘human’/exp
Web of Science	(TS=(oral microbiome) OR TS=(oral microbiota)) AND (TS=(diet) OR TS=(feeding behavior) OR TS=(food preferences) OR TS=(vegan) OR TS=(vegetarian) OR TS=(plant-based) OR TS=(mediterranean) OR TS=(DASH) OR TS=(ultra-processed) OR TS=(sugar) OR TS=(low-carbohydrate) OR TS=(ketogenic) OR TS=(high-protein) OR TS=(low-fat)) AND TS=(human)

**Table 2:** Main characteristics of the included studies.

Study	Design, Country	Sample	Population	Diet	Sample, Method	Key Findings
Daniele et al.; 2021 [31]	Cross-sectional; Italy	42	Adults; BMI ~22–23	Mediterranean vs. vegan	Saliva; 16S rRNA (specific regions not reported)	Alpha diversity: Mediterranean diet associated with a broader salivary microbial spectrum compared with vegan diet; Beta diversity: not clearly reported; Differential taxa: Mediterranean diet associated with higher abundance of *Prevotella* and *Neisseria subflava*, whereas *Lactobacillus* abundance was inversely associated with favorable metabolic parameters; Functional/metabolic findings: Mediterranean diet associated with higher basal metabolic rate and lower respiratory quotient.
De Filippis et al.; 2014 [11]	Cross-sectional; Italy	161	Adults; BMI > 18 (~22)	Omnivore vs. vegetarian vs. vegan	Saliva; 16S rRNA (V1–V3)	Alpha diversity: no significant differences among omnivorous, vegetarian, and vegan diets; Beta diversity: no significant differences in community structure according to diet; Differential taxa: no consistent diet-associated taxonomic signatures identified; Functional/metabolomic findings: salivary metabolome differed according to dietary pattern despite stable microbiota composition.
Hansen et al.; 2018 [12]	Cross-sectional; Denmark	160	Adults; BMI ~31	Vegan vs. omnivore	Saliva (fasting); 16S rRNA (V4)	Alpha diversity: no significant differences between vegans and omnivores; Beta diversity: subtle but significant differences in community structure according to diet; Differential taxa: vegans showed higher abundance of *Neisseria subflava*, *Haemophilus parainfluenzae*, *Rothia mucilaginosa*, *Campylobacter rectus*, and *Porphyromonas endodontalis*, while omnivores showed higher *Prevotella melaninogenica* and *Streptococcus* spp.; Functional/metabolic findings: functional microbial genomic profile differed between dietary groups.
Innocenti et al.; 2022 [30]	Cross-sectional (pilot); Italy	40	Adults; BMI ~23–24	Mediterranean diet adherence	Dental calculus; 16S rRNA (V3–V4)	Alpha diversity: not clearly reported, inter-individual variability observed in dental calculus microbiota; Beta diversity: Mediterranean diet adherence influenced microbial community composition; Differential taxa: diet-associated differences identified in dental calculus (e.g., *Prevotella*, *Eikenella*, and *Lentimicrobium* associated with higher Mediterranean adherence. Functional/metabolic findings: functional microbial pathways associated with dietary patterns.
Laiola et al.; 2020 [29]	Interventional; Italy	49	Adults; BMI ~21	Mediterranean diet intervention	Saliva (fasting); 16S rRNA (V3–V4)	Alpha diversity: no significant differences following Mediterranean diet intervention; Beta diversity: no significant clustering or overall community changes after intervention; Differential taxa: Mediterranean diet intervention resulted in a more favorable microbial profile associated with periodontal health (i.e., reduced *Porphyromonas gingivalis*, *Prevotella intermedia*, and *Treponema denticola*, and increased *Streptococcus cristatus* abundance); Functional findings: inflammatory status variations according to Mediterranean diet adherence and plant-based dietary intake.
Sun et al.; 2024 [32]	Cross-sectional; China	36	Adults; BMI information not reported	Vegan vs. seafood vs. red meat omnivores	Saliva (fasting); 16S rRNA (V3–V4)	Alpha diversity: differences in microbial composition observed among dietary groups; Beta diversity: vegetarian subjects showed distinct clustering compared with meat-based dietary groups; Differential taxa: vegetarians were enriched in *Neisseria*, whereas seafood- and red meat-based omnivores were enriched in *Streptococcus*; Functional/metabolomic findings: dietary groups showed distinct metabolic pathway profiles involving lipid, amino acid, carbohydrate, and nucleotide metabolism.

**Table 3:** Direction-of-effect summary table summarizing the main findings across included studies.

Study	Dietary Comparison	Alpha Diversity	Beta Diversity	Differential Taxa	Functional/Metabolomic Findings	Overall Interpretation
Daniele et al.; 2021 [31]	Mediterranean vs. vegan	↑ Greater microbial richness in Mediterranean diet	Not reported	↑ Higher Prevotella and Neisseria subflava in Mediterranean diet	↑ Favorable metabolic profile associated with Mediterranean diet	Mediterranean diet associated with specific microbial/metabolic features
De Filippis et al.; 2014 [11]	Omnivore vs. vegetarian vs. vegan	↔ No significant differences	↔ No significant differences	↔ No consistent taxa differences	↑ Diet-related metabolomic differences	Stable microbiota despite metabolomic variation
Hansen et al.; 2018 [12]	Vegan vs. omnivore	↔ No significant differences	↑ Subtle community differences	↑ Several taxa differed by diet	↑ Functional pathway differences	Mild diet-associated ecological shifts
Innocenti et al.; 2022 [30]	Mediterranean diet adherence	Not reported	↑ Community differences according to diet adherence	↑ Diet-associated taxonomic variation	↑ Functional pathway differences	Dietary adherence associated with ecological variation
Laiola et al.; 2020 [29]	Mediterranean diet intervention	↔ No significant differences	↔ No significant differences	↓ Periodontopathogenic taxa after intervention	↑ Associations with Mediterranean diet adherence	Reduction in periodontal-associated taxa
Sun et al.; 2024 [32]	Vegan vs. seafood vs. red meat omnivores	↑ Group differences reported	↑ Distinct clustering by diet	↑ Neisseria enriched in vegetarians; Streptococcus in omnivores	↑ Distinct metabolic pathways	Diet-associated taxonomic and metabolomic signatures

↑: increase; ↔: no changes; ↓: decrease.

## Data Availability

All data associated with this study are included in the article and Supplementary Material.

## Supplementary Materials

The following supporting information can be downloaded at: https://www.mdpi.com/article/10.3390/nu18111717/s1, Table S1: Detailed criteria for the risk of bias assessment and PRISMA checklist.
