# Supplementary material for: Oral Microbiota Characteristics in Relation to Different Dietary Patterns: A Systematic Review
Source: Nutrients. 2026 May 27;18(11):1717. doi: 10.3390/nu18111717 (PMC13258783; doi:10.3390/nu18111717)
Supplement: Supplementary file 1 [file nutrients-18-01717-s001.zip › nutrients-4287732-supplementary.pdf]

Supplementary material

Manuscript title: Oral microbiota characteristics in relation to different dietary patterns: a systematic review

Authors: Alessandro CHIESA, Luigi GENERALI, Andrea BUTERA, Tommaso FILIPPINI, Valentina LANTERI, and Federica VENERI.

Supplementary Table S1. Detailed criteria for the risk of bias assessment

Domain	Low risk	Moderate risk	High risk
**Research question**	The aim of the study is clearly stated; the research question is focused and appropriate	The aim of the study is vaguely stated; the research question is broad and explorative	The aim of the study and the research question are not stated
**Population**	Eligibility criteria are clearly stated; the population is adequately described	Data not reported regarding BMI and other anthropometrics	A priori eligibility criteria are not clearly specified
**Dietary exposure**	validated assessment of dietary habits (e.g. validated questionnaires with adherence score reported); controlled intervention	Self-reported dietary questionnaires, with no adherence scores	Assessment method not reported
**Microbiome assessment**	Standard validated sequencing methods, with adequate technical details	Validated microbial analytical methods; no sequencing or missing technical details	Assessment method not reported
**Confounders**	Adequate control of confounders (adjustments, sensitivity analyses or standardization for lifestyle/oral hygiene/smoke/BMI/alcohol consumption)	Partial control of confounders (limited adjustments or sensitivity analyses for lifestyle/oral hygiene/smoke/BMI/alcohol consumption)	Potential confounders not reported or not considered (lack of population details regarding lifestyle/oral hygiene/smoke/BMI/alcohol consumption)
**Sample size**	Adequate, representative sample size (e.g., n>= 50 participants per dietary group)	Moderately representative sample size (e.g., n= 20-50 participants per dietary group)	Small non-representative sample size (e.g., n<20 participants per dietary group)
